# Double autophagy modulators reduce 2-deoxyglucose uptake in sarcoma patients

**DOI:** 10.18632/oncotarget.5060

**Published:** 2015-09-07

**Authors:** Mau-Shin Chi, Cheng-Yen Lee, Su-Chen Huang, Kai-Lin Yang, Hui-Ling Ko, Yen-Kung Chen, Chen-Han Chung, Kuang-Wen Liao, Kwan-Hwa Chi

**Affiliations:** ^1^ Department of Radiation Therapy and Oncology, Shin Kong Wu Ho-Su Memorial Hospital, Taipei, Taiwan; ^2^ Department of Nuclear Medicine and PET Center, Shin Kong Wu Ho-Su Memorial Hospital, Taipei, Taiwan; ^3^ Institue of Molecular Medicine and Bioengineering, National Chiao-Tung University, Hsinchu, Taiwan; ^4^ School of Medicine and Institute of Biomedical Imaging and Radiological Sciences, National Yang-Ming University, Taipei, Taiwan

**Keywords:** hydroxychloroquine, sirolimus, soft tissue sarcoma

## Abstract

**Rationale:**

According to the metabolic symbiosis model, cancer stromal fibroblasts could be hijacked by surrounding cancer cells into a state of autophagy with aerobic glycolysis to help provide recycled nutrients. The purpose of this study was to investigate whether combined treatment with the autophagy inhibitor: hydroxychloroquine (HCQ) and the autophagy inducer: sirolimus (rapamycin, Rapa) would reduce glucose utilization in sarcoma patients.

**Methods:**

Ten sarcoma patients who failed first-line treatment were enrolled in this study. They were treated with 1 mg of Rapa and 200 mg of HCQ twice daily for two weeks. The standardized uptake values (SUV) from pretreatment and posttreatment [18F]-fluorodeoxyglucose positron emission tomography (FDG PET) scans were reviewed, and changes from the baseline SUVmax were evaluated.

**Results:**

Based on FDG PET response criteria, six patients had a partial response; three had stable disease, and one had progressive disease. Nevertheless, none of them showed a reduction in tumor volume. The mean SUVmax reduction in the 34 lesions evaluated was − 19.6% (95% CI = −30.1% to −9.1%), while the mean volume change was +16.4% (95% CI = +5.8% to + 27%). Only grade 1 toxicities were observed. Elevated serum levels of lactate dehydrogenase were detected after treatment in most metabolic responders.

**Conclusions:**

The results of reduced SUVmax without tumor volume reduction after two weeks of Rapa and HCQ treatment may indicate that non-proliferative glycolysis occurred mainly in the cancer associated fibroblast compartment, and decreased glycolytic activity was evident from Rapa + HCQ double autophagy modulator treatment.

## INTRODUCTION

The reported worldwide incidence rate of soft tissue sarcoma ranges from 1.8 to 5 per 100,000 people per year [1]. Multimodality treatment is often provided to patients with primary disease, whereas chemotherapy is given to those with metastatic disease. Unfortunately, sarcoma is usually chemo-insensitive. In addition to the intrinsic chemo-insensitivity of sarcoma cells, the interaction between a tumor and its microenvironment, which triggers chemo-resistance, might be of importance [2]. Cancer-associated fibroblasts (CAFs), which constitute a substantial volume in a tumor microenvironment, are closely associated with invasiveness, drug resistance, and metastasis to fibroblasts, endothelial cells, or adipose tissue [3, 4].

Martinez-Outschoorn *et al*. proposed the autophagic tumor stroma model and reported that cancer cells could be seen as “metabolic parasites” for using oxidative stress to extract recycled nutrients from adjacent stromal cells [5, 6]. CAFs could produce high-energy nutrients (e.g., lactate and ketones) that fuel mitochondrial biogenesis and oxidative metabolism in cancer cells. This novel energy transfer mechanism is termed the “reverse Warburg effect” [7, 8]. Autophagy in the tumor stroma serves as a “battery” to fuel tumor growth, progression, and metastasis, often independent of angiogenesis [5]. Hydroxychloroquine (HCQ) or other autophagy/lysosome inhibitors may be useful to therapeutically restore the “normal” CAFs by blocking their state of hyper-autophagy. Furthermore, induction of sarcoma cell autophagy with sirolimus (rapamycin, Rapa) could prevent them from using recycled nutrients [5]. Thus, concurrent administration of the two above-mentioned agents might block the metabolic parasite relationship within a sarcoma tumor microenvironment [5, 8]. Such a strategy may effectively “starve” cancer cells, rendering them more vulnerable to cytotoxic therapy. The metabolic modulation and change in bioenergetic homeostasis of the tumor microenvironment could affect mitochondrial electron transport complexes and glycolysis-related pathways in cancer cells, thus altering reactive oxygen species and adenosine triphosphate synthesis, and decreasing their survival capability under chemotherapy stress [9, 10].

Since CAFs usually have the largest increase in glucose uptake, [18F]-fluorodeoxyglucose positron emission tomography (FDG PET) of tumors might specifically detect the tumor stroma rather than the tumor cells [11]. The purpose of this study was to demonstrate that a decrease in glucose metabolism could mirror the attenuating metabolic-parasite relationship between CAFs and sarcoma cells by Rapa and HCQ treatment.

## RESULTS

The primary end point of this study was the SUVmax change after two weeks of Rapa and HCQ treatment. The secondary end point was the response rate after eight weeks of treatment. However, most patients discontinued treatment before response assessment because of progression of disease and slow recruitment of cases (13 patients from August 2012 to June 2014), so the study was closed prematurely. Ten patients were evaluable by sequential [18F]-FDG PET images at baseline and after two weeks of treatment. Patient characteristics of these ten patients are shown in Table 1. All of the patients tolerated Rapa and HCQ treatment well with only mild grade 1 adverse events and were observed after a median of two weeks treatment (range two to four weeks) (Table 2).

**Table 1:** Patient characteristics (*n* = 10)

Variables	*n*	%
**Sex**		
Male	5	50
Female	5	50
**Pathology**		
Liposarcoma	2	20
Leiomyosarcoma	2	20
Osteosarcoma	1	10
Angiosarcoma	1	10
Fibrosarcoma	1	10
Sarcomatoid carcinoma	1	10
Malignant fibrous histiocytoma	1	10
Endometrioid stromal sarcoma	1	10
	**Median**	**Range**
Age (years)	63	17–80

**Table 2:** Toxicities (*n* = 10)

Adverse effect	Grade 1 (*n*)	%
Mucositis	0	0
Skin rash	2	20
Hair loss	0	0
Nausea	3	30
Diarrhea	1	10
Constipation	1	10
Dyspnea	0	0
Somnolence	0	0
Asthenia	0	0
Fever	0	0

**Figure 1:**
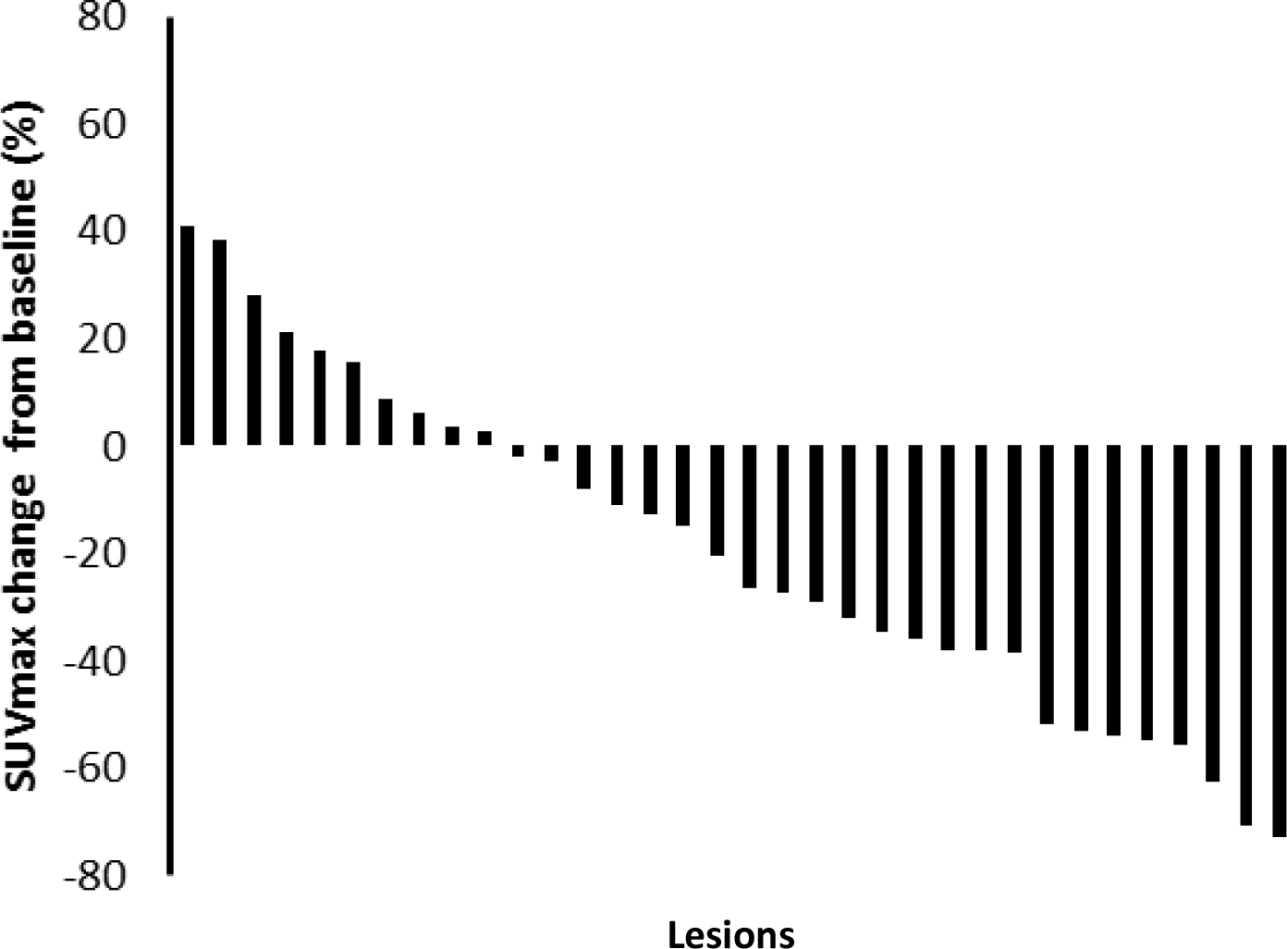
A waterfall plot of posttreatment changes in SUVmax from baseline for thirty-four evaluable lesions

**Figure 2:**
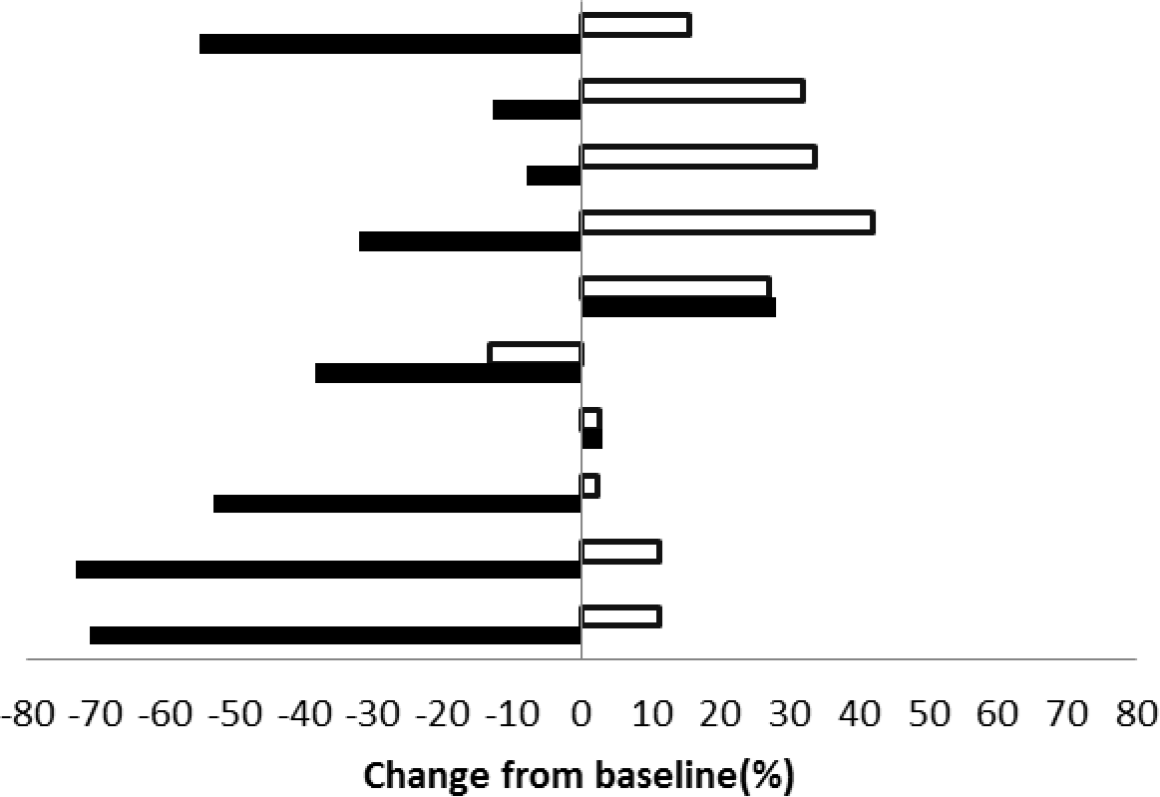
Maximum post treatment SUVmax versus tumor volume changes from baseline for each enrolled patient (■ indicates SUVmax change, □ indicates tumor volume change).

**Figure 3:**
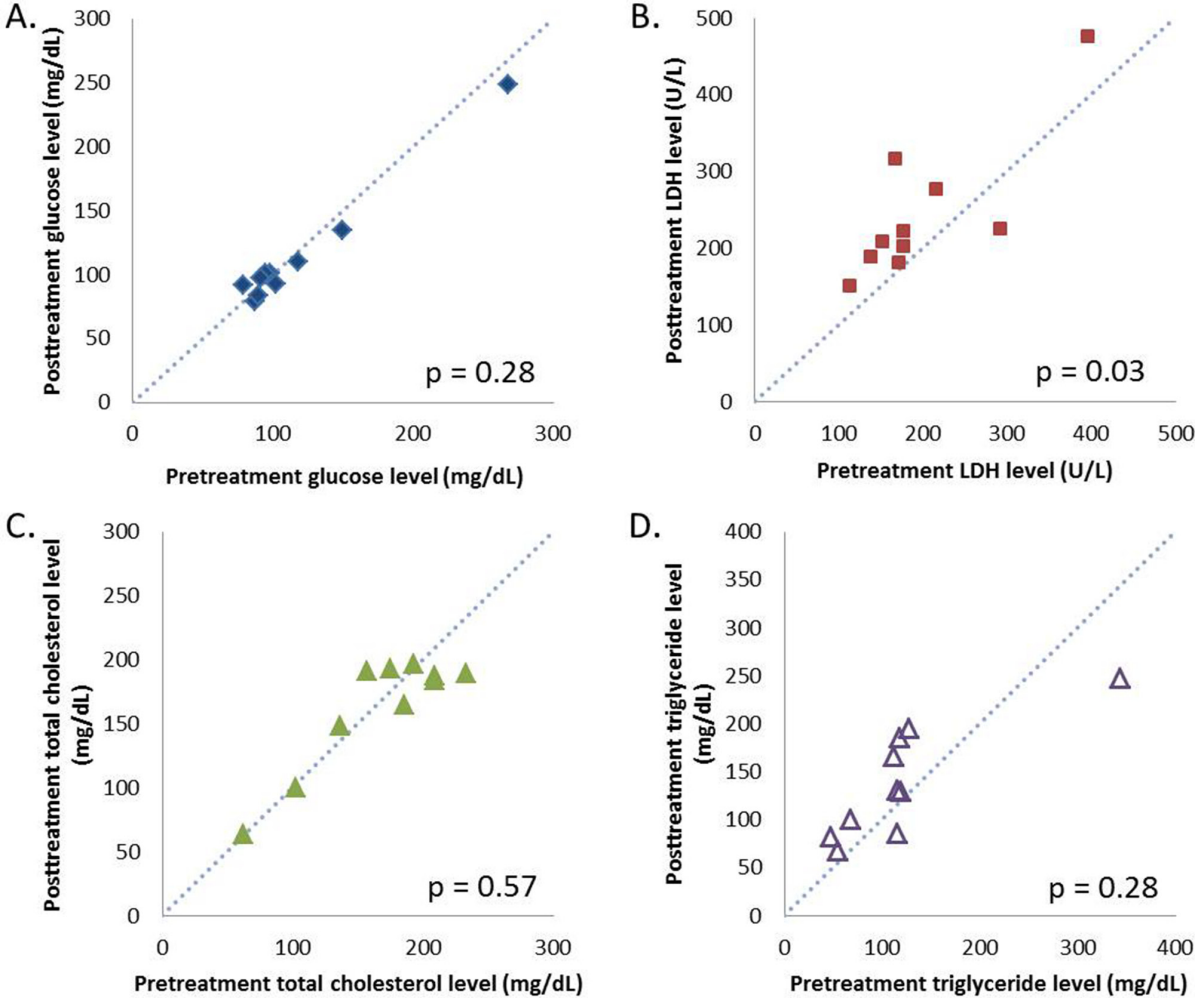
Calibration plots of fasting plasma glucose A. lactate dehydrogenase B. total cholesterol C. and triglyceride D. levels before and after two weeks of hydroxychloroquine and sirolimus treatment

**Figure 4:**
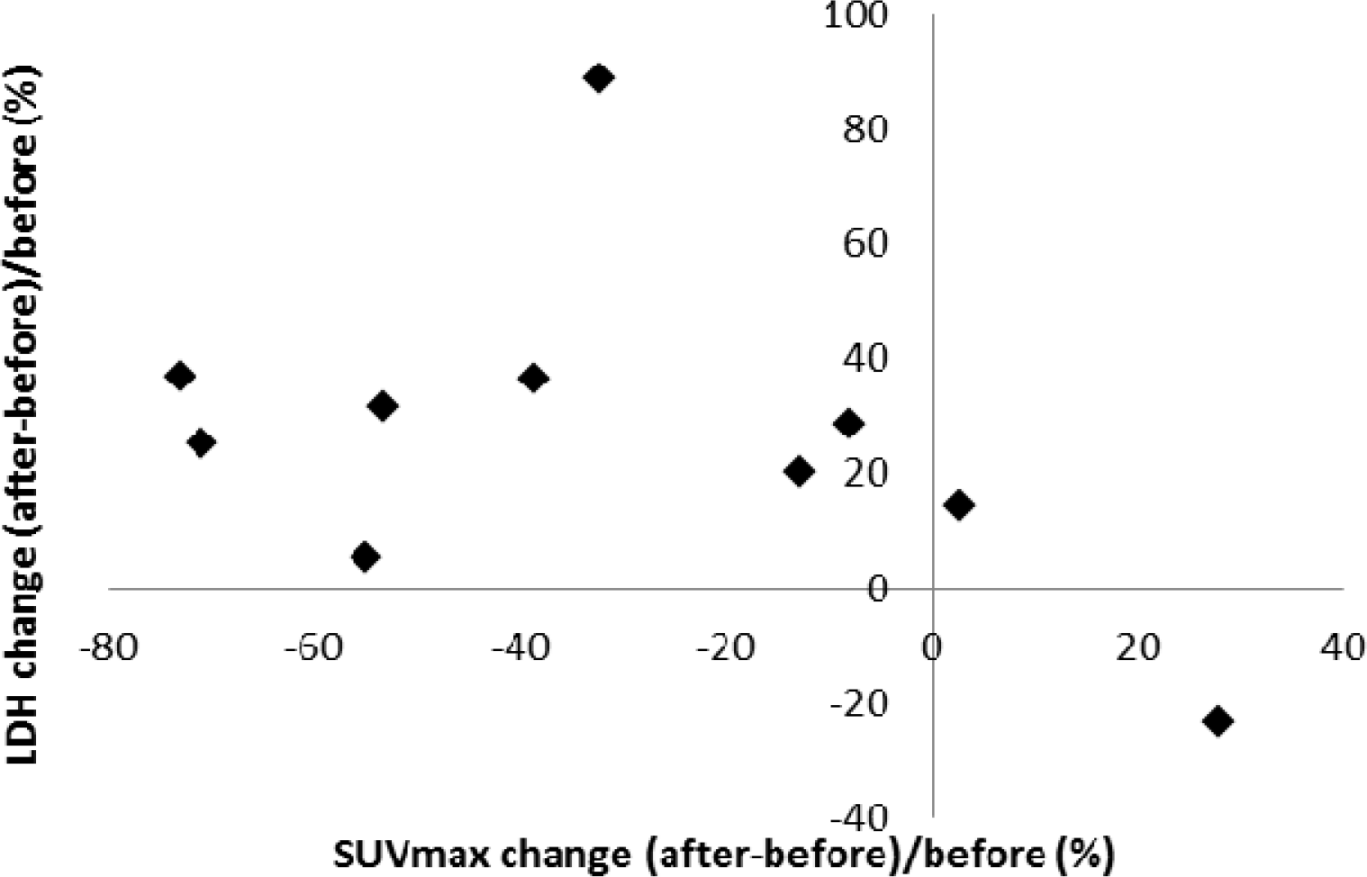
Correlation between SUVmax and lactate dehydrogenase level changes after two weeks of hydroxychloroquine and sirolimus treatment

There were 34 lesions evaluable for changes of SUVmax; these changes are presented in Figure 1. The mean baseline SUVmax was 13.5 (95% confidence interval [CI] = 10.1–16.9), and the mean posttreatment value was 9.9 (95% CI = 7.5–12.3; *p* < 0.003). The mean reduction in SUVmax was − 19.6% (95% CI = − 30.1 to − 9.1%), and the median was − 23.7% (interquartile range, − 41.8% to 4.1%). The changes of SUVmax versus volume changes form the ten enrolled patients are shown in Figure 2. The mean change of SUVmax was −31.3% (95% CI = −51.9% to – 10.6%) while the mean volume change on the same lesion was + 16.4% (95%CI = + 5.8% to + 27.0%). The disparity between glucose consumption rate and proliferation need in the tumors suggested a metabolic reprogramming process might have occurred. According to the EORTC 1999 criteria, six patients had a partial response, three had a stable disease, and one had a progressive disease. The biochemical profile changes before and after two weeks of Rapa and HCQ treatment are shown in Figure 3. Overall, there were no significant changes in the fasting plasma glucose, total cholesterol, or triglyceride levels. However, as shown in Figure 3B, the lactate dehydrogenase (LDH) level was significantly increased after treatment. Interestingly, most patients with elevated LDH levels were metabolic responders (Figure 4).

## DISCUSSION

This study demonstrated for the first time that a metabolic symbiosis relationship between CAFs and sarcoma cells might be altered by Rapa and HCQ treatment. An inhibition of glycolysis within the tumors without tumor growth was noted.

**Figure 5:**
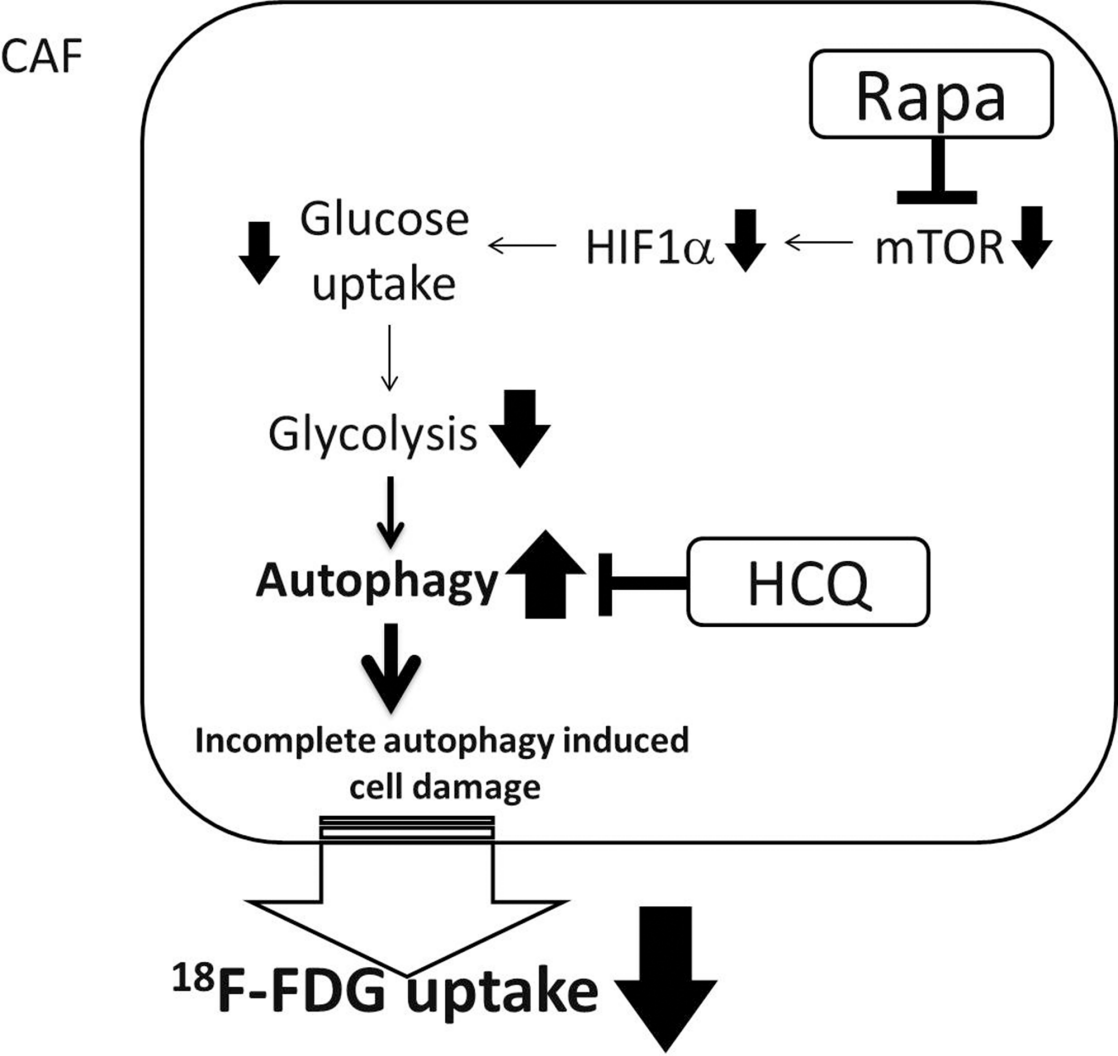
Synergistic effect of Rapa and HCQ combination CAF cells are glycolytic-dependent cells. The combined use of Rapa and HCQ has synergistic effect on reducing glucose consumption rate.

**Figure 6:**
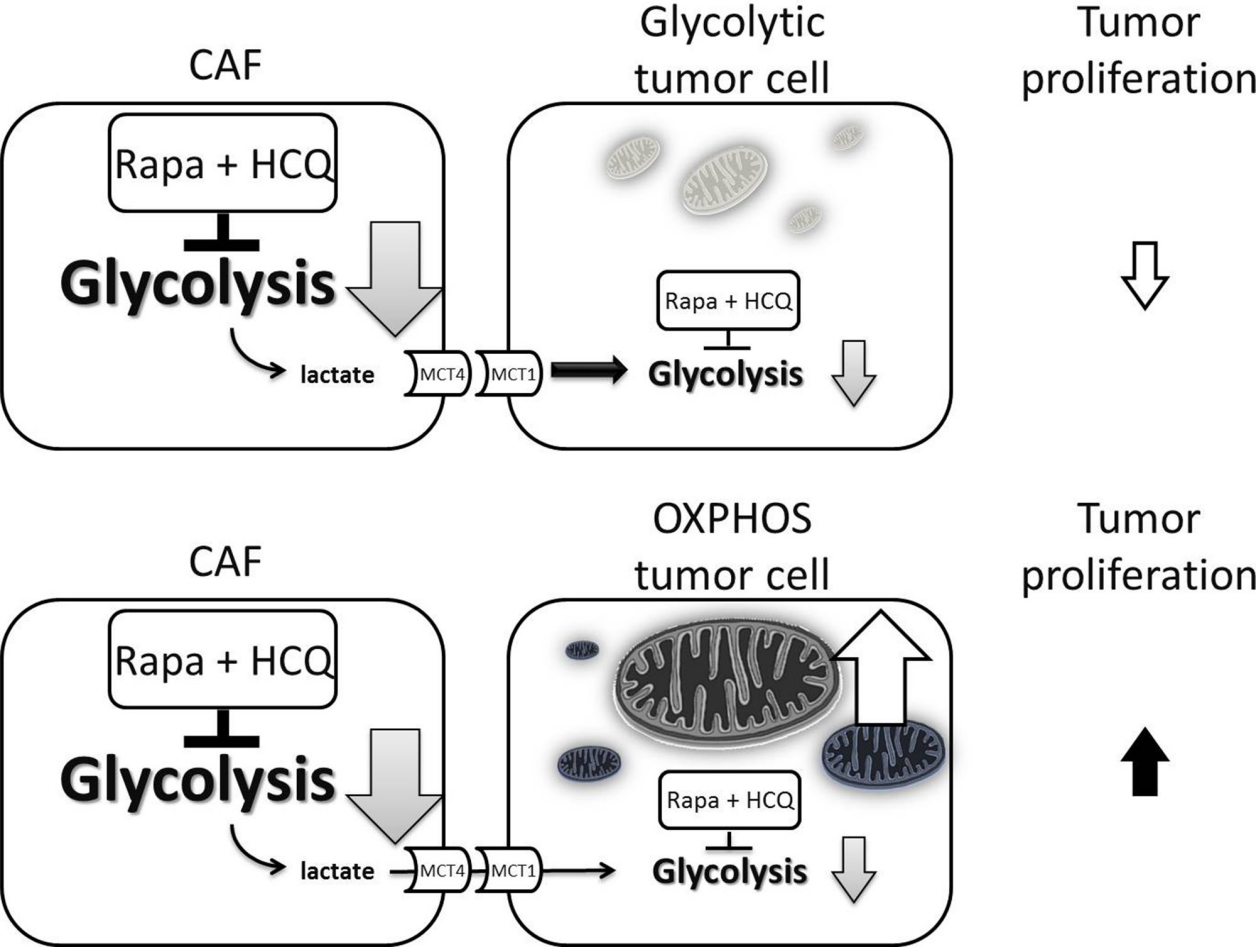
Model of uncoupling energy transfer within sarcoma tumor Rapa and HCQ combination will inhibit the aerobic glycolysis and reduce the energy transfer from CAFs to OXPHOS sarcoma cells and glycolytic sarcoma cells. The decreased lactate supply has little effect on OXPHOS sarcoma cells due to their independent energy sources, their mitochondria.

According to the metabolic symbiosis theory, cancer cells induce oxidative stress in adjacent fibroblasts, resulting in the onset of a myofibroblastic pro-autophagic phenotype [13]. This pro-autophagic phenotype in fibroblasts leads to a loss of mitochondria via autophagy, forcing CAFs to undergo aerobic glycolysis and provide lactate as energy source for cancer cells. Autophagy in the tumor stroma is also used by adjacent epithelial cells to fuel tumor growth via oxidative mitochondrial metabolism [14, 15]. The autophagy inhibitor chloroquine (or HCQ) blocks the process in CAFs and stops the energy flow to sarcoma cells, while the autophagy inducer, Rapa, inhibits mammalian target of rapamycin (mTOR) signaling and induces autophagy in cancer cells to reduce tumor growth rate. The so-called autophagy paradox by concurrent administration of an autophagy inducer and inhibitor could be explained by the two-compartment model in which the two drugs were suggested to have different target preferences [16]. However, Rapa and HCQ combination may have a synergistic effect on the same target. As shown in Figure 5, the inhibition of the mTOR signal by RAPA decreased hypoxia-inducible factor 1-alpha (HIF-α) and glucose uptake and therefore decreased glycolysis. The decreased glycolysis activated more autophagy in glycolysis-dependent cells and rendered them more vulnerable to HCQ treatment by accumulation of wasted products in lysosomes. The decreased glycolytic activity without a concomitant decreased proliferation rate suggested that the Rapa and HCQ combination might not be able to block the proliferative signals in sarcoma cells. Figure 6 shows the coexistence of oxidative phosphorylation (OXPHOS) sarcoma cells and glycolytic sarcoma cells, similar to the metabolic coupling model proposed from osteosarcoma [17, 18]. Although the Rapa and HCQ combination decreased the glycolytic activity, the decreased lactate supply may have compensated by stimulating the OXPHOS function in the mitochondria-rich sarcoma cells, based on the observed tumor proliferation and decreased glycolytic activity.

The inhibition of glucose metabolism assessed by [18F]-FDG PET without a decrease in tumor proliferation after Rapa plus HCQ treatment in sarcoma patients is somewhat surprising, because reduction of both SUVmax and SUV (mean) are generally regarded as indicative of decreased tumor viability. The observed decrease in glucose consumption on tumor area may have been largely due to the tumor microenvironment and therefore independent of tumor viability. This finding was novel in certain aspects. Firstly, this is the first report on the effect of combined Rapa and HCQ on glucose metabolism in cancer patients. To our knowledge, no publication is available regarding the effect of combined double autophagy modulators on glucose metabolism measured by [18F]-FDG PET in cancer patients. A Rapa analogue, everolimus, has been reported to decrease glucose uptake in few type of cancers [19, 20]. The decrease in glucose uptake by everolimus has not been linked to the inhibition of glycolysis rather than cytotoxicity in any study. Secondly, our results provide functional image evidence of the metabolic parasite relationship proposed by Lisanti's group, namely that catabolic fibroblasts donate the necessary fuels to anabolic cancer cells [7, 8]. The non-proliferative and mitochondrial-poor stromal cells are probably dominant in the sarcoma tumor microenvironment, where CAFs usually have the largest increase in glucose uptake. [9] Thirdly, the Martinez-Outschoorn's theory of “battery-operated tumor growth” might not be reversed by Rapa and HCQ treatment because the discrepancy of decreased glucose uptake with increased tumor volume may indicate that proliferative and mitochondrion-rich cancer cells still thrive after autophagy modulation therapy.

Rapa and newer generation mTOR inhibitors have limited clinical activity, except in certain tumor types with heavy dependence on mTOR [20, 21]. Sarcoma is not one of them. Recently, either a Rapa analogue or HCQ has been used as an adjunctive anti-cancer agent in clinical trials [22–24]. The median decrease in SUVmax of −23.7% by Rapa and HCQ treatment in our patient cohort cannot be considered an effective sarcoma treatment modality based on the current results. Since cancer cells use autophagy as a pro-survival mechanism during chemotherapy, we hypothesized that a strategy of combined Rapa, HCQ, and chemotherapy as a triplet combination should be more synergistic than only Rapa and HCQ doublet combination and might reverse drug resistance for the following reasons: (1.) The use of Rapa and chemotherapy might promote cancer cells (including sarcoma) to a higher autophagy state, while HCQ blocks their final autolysosome pathway and switches the pro-survival to pro-death process as an example of synthetic lethality [25], (2.) Systemic HCQ might block the vicious cycle of energy supply from the tumor microenvironment through the inhibition of CAF's autophagy, and (3.) The metabolic uncoupling of oxidative cancer cells from glycolytic stroma by Rapa and HCQ may render cancer cells more vulnerable to mitochondria-targeted drugs or oxidative stresses.

As shown in Figure 4, LDH levels significantly increased after treatment, which might indicate a compensatory process when aerobic glycolysis was inhibited by Rapa and HCQ. LDH levels might be used as surrogate biomarkers in future clinical trials. It has been reported that a dose-dependent rise in LDH after treatment with everolimus and octreotide correlated with better progression-free survival on neuroendocrine tumors. [26] A rise in LDH in association with mTOR inhibition has been reported to be associated anti-angiogenesis associated hypoxia [27]. An everolimus and chloroquine combination has also been reported to inhibit angiogenesis [28]. Recent studies showed that the endothelial cells were one of the most critical tissues depending on aerobic glycolysis [29]. The rise in LDH shortly after treatment with autophagy modulators provide more evidence to suggest that a direct anti-glycolysis effect from the Rapa and HCQ doublet combination occurs in the tumor microenvironment.

## MATERIALS AND METHODS

### Patients

This study was reviewed and approved by our institutional review board. All patients provided written informed consent before participation. It was registered with ClinicalTrials.gov under registration No. NCT01842594. Patients with histologically confirmed sarcoma who failed first-line treatment were eligible. In addition, the eligibility criteria included at least one measurable lesion (>2 cm), an Eastern Cooperative Oncology Group performance status of 0–2, as well as adequate hepatic, renal, and hematological functions (levels of serum bilirubin ≤ 25 mg/dL, aspartate aminotransferase ≤ 5 × institutional upper limit of normal, serum creatinine ≤ 2.0 mg/dL, absolute neutrophil count ≥ 1.5 × 10^9^/L, and platelet count ≥ 100 × 10^9^/L). Patients were required to be free from any previous chemotherapy for more than five weeks before entering this study. Those with clinically significant cardiovascular diseases such as uncontrolled hypertension, myocardial infarction, unstable angina, or congestive heart failure of New York Heart Association grade II and higher were excluded.

### Procedures

All of the patients underwent a physical examination and laboratory assessment of hematological, hepatic, and renal functions before entering the study. A baseline whole-body [18F]-FDG PET was performed before therapy initiation. Patients received 1 mg of Rapa and 200 mg of HCQ twice a day before meal. A second [18F]-FDG PET was performed two weeks later. Toxicity was assessed according to the National Cancer Institute Common Terminology Criteria version 3.0. No dose modification was allowed.

All of the PET scans were conducted using a Siemens Biograph mCt PET/CT scanner (Siemens Medical Solutions Inc., Molecular Imaging, IL, USA) with extended axial field of view (TrueV). The patients were required to fast for at least eight hours before the PET/CT scan. PET attenuation correction factors were calculated from CT images using low-dose CT (120 keV and 20 mAs) in shallow inspiration. The scanner had an average spatial resolution of 4.4 mm at 1 cm and 5.0 mm at 10 cm from the transverse field of view (FOV) and a maximum sensitivity of 8.1 kcps/ MBq at center of FOV. Its axial FOV was 21.6 cm. After intravenous administration of 185 MBq (5 mCi) of [18F]-FDG, PET images were acquired for three minutes per bed position. The uptake period between the FDG injection and the beginning of PET scan was 60 ± 10 minutes (range, 50–70 minutes). The PET/CT scan started from the head and moved toward the pelvis; a head pillow and knee cushion were used to render positioning comparable in both scanners. Images were reconstructed via proprietary Siemens HD PET software using the iterative TrueX and time of flight ordered-subsets expectation maximization (TOF OSEM) method. They were displayed in three orthogonal projections and as a whole-body maximum-pixel-intensity reprojection image for visual interpretation.

SUVs were calculated for all lesions. Regions of interest (ROI) were contoured to represent tumors (>2 cm) and organs (lungs, spleen, and liver) on all transaxial and coronal slices. ROIs were normalized for injection dose and body weight, and the maximum voxel value was recorded for each region or organ. The highest SUV measured with increased uptake was considered the SUVmax. Correlative diagnostic CT examinations were used for accurate localization. The most intense uptake at baseline was identified as the index lesion and evaluated for treatment response.

Two medical physicists independently analyzed the changes in SUVmax between baseline and post treatment PET scans. Metabolic response after two weeks of treatment was evaluated according to the European Organization for Research and Treatment of Cancer (EORTC 1999) guidelines [12]. A complete response was defined as the complete resolution of [18F]-FDG uptake within the tumor volume. A partial response was defined as a reduction of 25% or greater in tumor [18F]-FDG uptake, whereas stable disease was defined as an increase or decrease in [18F]-FDG uptake of less than 25%. Progressive disease was defined as an increase in [18F]-FDG uptake of 25% or higher, or as the appearance of a new [18F]-FDG uptake focus.

The CT images were forward to the Pinnacle treatment planning system, version 9.8 (Philips, Fitchburg, WI, USA) where tumor sizes were measured on a digitalized image. An independent radiation oncologist ensured that the same lesions on SUVmax were measured and evaluated at pretreatment and on follow-up scans.

### Statistical analysis

Percent changes from baseline SUVmax and tumor volume after Rapa and HCQ treatment were plotted as a waterfall chart. Differences in the results of comparative tests were determined by *t*-test and considered significant if the two-sided *p* < 0.05. All statistical analysis was performed using the Statistical Application System software, SAS Version 9.1.3(SAS institute, Inc., Cary, NC, USA).

## CONCLUSIONS

The cancer metabolism pathway compartmentalization model (oxidative vs glycolytic) is still in its infancy due to limited clinical methods for direct study. The combination treatment with Rapa and HCQ in sarcoma patients by [18f]-FDG-PET revealed a reduction of 2-deoxyglucose uptake while increasing tumor volume, which fits the catabolic tumor fibroblasts and anabolic cancer cells model well. The reversal of “reversed Warburg effect” in CAFs by the combination of autophagy inducers (Rapa) and autophagy inhibitor (HCQ) may offer opportunities for treatment strategies targeted on the deregulated metabolism in tumor microenvironment.
